# A Morphological and Behavioral Study of Demyelination and Remyelination in the Cuprizone Model: Insights into APLNR and NG2+ Cell Dynamics

**DOI:** 10.3390/ijms252313011

**Published:** 2024-12-03

**Authors:** Boycho Landzhov, Lyubomir Gaydarski, Stancho Stanchev, Ivanka Kostadinova, Alexandar Iliev, Georgi Kotov, Pavel Rashev, Milena Mourdjeva, Despina Pupaki, Nikola Stamenov

**Affiliations:** 1 Department of Anatomy, Histology and Embryology, Medical University of Sofia, 1431 Sofia, Bulgaria; landzhov_medac@abv.bg (B.L.); stanchev_1989@abv.bg (S.S.); dralexiliev@abv.bg (A.I.); nikola.stamenov1@gmail.com (N.S.); 2 Department of Pharmacology, Pharmacotherapy and Toxicology, Medical University of Sofia, 1000 Sofia, Bulgaria; i.kostadinova@pharmfac.mu-sofia.bg; 3 Department of Rheumatology, Clinic of Rheumatology, University Hospital “St. Ivan Rilski”, Medical University of Sofia, 1431 Sofia, Bulgaria; gn_kotov@abv.bg; 4 Institute of Biology and Immunology of Reproduction, Bulgarian Academy of Sciences, 1113 Sofia, Bulgaria; pavel_rashev@abv.bg (P.R.); milena_mourdjeva@abv.bg (M.M.); poupaki_desi@abv.bg (D.P.)

**Keywords:** apelin receptor (APLNR), neuronal/glial antigen 2 (NG2), subventricular zone (SVZ), cuprizone model, passive avoidance test, rotarod test

## Abstract

Multiple sclerosis (MS) is a chronic neurodegenerative disorder involving demyelination. The cuprizone model is commonly used to study MS by inducing oligodendrocyte stress and demyelination. The subventricular zone (SVZ) plays a key role in neurogenesis, while the neuronal/glial antigen 2 (NG2) is a marker for immature glial cells, involved in oligodendrocyte differentiation. The apelin receptor (APLNR) is linked to neurogenesis and behavior modulation. This study explores the role of APLNR in NG2-positive cells during de- and remyelination phases in the experimental cuprizone mouse model. Thirty male C57BL/6 mice were divided into control (not treated), demyelination (5 weeks cuprizone administration), and remyelination (5 weeks cuprizone administration + 5 weeks recovery) groups. Histological examinations, immunohistochemistry, and immunofluorescence on serial coronal sections were conducted to evaluate corpus callosum (CC) morphology and APLNR and NG2 expression in the SVZ, in addition to behavioral assessments. The histological analysis showed a significant reduction in the CC’s thickness and area after five weeks of cuprizone exposure, followed by recovery five weeks post-exposure. During the demyelination phase, APLNR-expressing cells peaked while NG2-positive cells decreased. In the remyelination phase, APLNR-expressing cells declined, and NG2-positive cells increased. Confocal microscopy confirmed the co-localization of NG2 and APLNR markers. Statistically significant differences were observed across experimental groups. Correlation analyses highlighted associations between APLNR/NG2 cell counts and CC changes. Behavioral tests revealed impaired motor coordination and memory during demyelination, with gradual recovery during remyelination. Significant changes in the CC structure and the number of APLNR and NG2-positive cells were observed during de- and remyelination, suggesting that NG2-positive cells expressing APLNR may play a key role in remyelination.

## 1. Introduction

MS is a chronic immune-mediated disease accompanied by chronic inflammation, progressive demyelination, gliosis, and neurodegeneration [1]. MS’s key pathological features include immune cell infiltration, blood–brain barrier disruption, gliosis, neuroaxonal degeneration, oligodendrocyte damage, and demyelination. These processes are initiated by autoimmune T- and B-lymphocyte interactions with myelin antigens and influenced by intrinsic brain cytodegenerative processes, leading to the progressive loss of oligodendrocytes, the degradation of the myelin sheaths, and astrogliosis [2,3,4]. To explore various aspects of MS pathology, several animal models are used, such as the experimental autoimmune encephalomyelitis (EAE) model, Theiler’s Murine Encephalomyelitis Virus (TMEV) model, the lysophosphatidylcholine (LPC) or lysolecithin model, and the cuprizone model [5]. The cuprizone model offers several advantages over the other mentioned experimental models. This model induces early oligodendrocyte stress in young mice (8-week-old C57BL/6 strain) by disrupting mitochondrial function due to copper deficiency [6,7]. This stress involves ferroptosis and endoplasmic reticulum responses [8,9]. While the exact mechanisms of oligodendrocyte degeneration are unclear, research suggests that a reactive cuprizone–copper complex may be responsible for affecting various cells in the central nervous system (CNS) [10]. Because of this, the cuprizone model allows for a more in-depth study of demyelination and remyelination processes in isolation from immune system involvement [6]. This is crucial for understanding the intrinsic mechanisms of oligodendrocyte injury and repair, as cuprizone toxicity primarily affects oligodendrocytes through oxidative stress rather than immune-mediated damage [6]. Acute cuprizone exposure leads to complete demyelination in specific brain regions, followed by microglial and astrocytic proliferation and axonal damage [11]. Cuprizone can induce a highly reproducible pattern of demyelination within a specific timeframe (4–6 weeks), allowing for precise experimental control. This contrasts with EAE, where variability in immune response can lead to inconsistent results [12]. Another advantage of the cuprizone model is its minimal impact on the peripheral nervous system (PNS). This allows for a better focus on CNS pathology without the confounding effects of systemic inflammation and PNS injury [6]. Endogenous remyelination occurs after acute exposure but is delayed with prolonged cuprizone administration, termed chronic demyelination, affecting multiple brain areas beyond the CC [13]. Those processes can be tracked by measuring the thickness of the CC at the level of the anterior commissure and used as histomorphometric parameters of demyelination, followed by remyelination [5].

The development of the neurons and macroglia in the CNS occurs in two main areas known as the ventricular and the SVZ. The SVZ is a germinal region around the lateral ventricle divided into smaller inner and more significant outer portions. The borders of the SVZ are the septum pellucidum (medially), striatum (laterally), and CC (dorsally) [14]. This zone is crucial for the differentiation of cortical projection neurons, interneurons, and glial cells [15]. Neuronal stem cells within the SVZ produce neuroblasts, which travel through the rostral migratory stream (RMS) to the olfactory bulb (OB), where they mature into different types of neurons and become part of existing neuronal circuits [16]. Additionally, neuronal stem cells in the postnatal SVZ give rise to astrocytes and oligodendrocytes [17]. 

The expression of NG2 within the CNS is mainly confined to immature glial cells, known as NG2-positive progenitor cells, and pericytes [18,19]. NG2-positive (NG2+) progenitor cells are usually described as an oligodendrocyte precursor cell type [20]. In addition, NG2+ progenitor cells may also differentiate into astrocytes [20]. NG2+ cells are ubiquitously found in the developing and adult CNS in white and gray matter, including the SVZ [21]. They are recognized as the fourth primary glial cell type in the mature CNS and constitute 2–8% of the whole cell population in the mature CNS [22].

The interaction between neurons and NG2+ cells includes excitatory and inhibitory neuron–glia synapses and suggests the potential role of NG2+ cells in various signaling pathways in the CNS [23]. The developmental origin of NG2+ progenitor cells includes medial, lateral, and/or caudal ganglionic eminence and the postnatal cerebral cortex [24]. The SVZ is considered a primary source for NG2+ cells during postnatal development [25]. Under pathological conditions, NG2+ cells show altered morphology, including hypertrophy, more pronounced immunohistochemical labeling for NG2 proteoglycan, and an increased number of outgrowths [26]. The increased NG2+ cell density seems to have controversial effects in the injured area, varying from the inhibition of axonal regeneration to a supporting function for axonal growth cones [27,28]. The reactivity of NG2+ cells in neurodegenerative diseases such as MS includes the prominent proliferation and formation of myelinating oligodendrocytes near the lesions [20]. While NG2+ cells show pronounced efficiency in remyelination in case of acute injury, these cells have limited capacity during chronic demyelination [29].

The apelinergic system consists of a G-protein coupled receptor known as the APLNR and its two endogenous peptide ligands, apelin and elabela [30]. Apelin is an essential peptide in regulating energy balance and contributes to cardiovascular, renovascular, gastrointestinal, neuroendocrine, and immune functions, body mass, and temperature [31,32,33]. In mammals, the apelinergic system is involved in neurogenesis, affects the activity of the pituitary gland and plays a role in controlling blood pressure [33,34,35,36,37]. It seems that apelin has a higher expression compared to APLNR in the telencephalon [38]. The expression of apelin and APLNR in the CNS includes limited areas of the cerebral cortex, supraoptic and paraventricular nuclei of the hypothalamus, dentate gyrus, caudate nucleus, hippocampus proper, preoptic area, brainstem, and spinal cord [36,39]. Moreover, Chongtham et al. 2020 described the expression of APLNR in the SVZ in primates [35]. It is well known that the subgranular zone of the dentate gyrus and the SVZ serve as the main niches for postnatal neurogenesis, and the apelinergic system may have a potential role in this process [40]. Chongtham et al. 2020 reported that the expression of APLNR in the anterior SVZ refers to neuronal progenitor cells [35]. Although apelin and APLNR are expressed in neurons, the apelinergic system is not evident in microglia. While APLNR is also found in oligodendrocytes and astrocytes, apelin is not detected in astrocytes [41,42].

Several studies have shown that rodents fed with cuprizone displayed an impairment in memory as evident by different behavior paradigms like Y-maze, novel object recognition tests [43], and passive avoidance tests [44]. On the other hand, the literature data reveal that progenitors and mature oligodendrocytes contribute to learning and memory by influencing synaptic transmission, axon excitability, and myelination to modify the impulse conduction speed [45]. Among the various functional deficits, gait and coordination abnormalities are commonly observed in patients with MS. Motor function is mediated by a complex neural network that originates in the cortex and terminates in the skeletal muscles. Several methods exist to determine gait abnormalities in small rodents, yet the rotarod paradigm is the most frequently used method to test the motor performance alterations in the cuprizone model [46].

Based upon the literature data regarding the role of the NG2+ cells in the processes of demyelination and remyelination, the present study strives to investigate the role of APLNR in NG2+ cells during de- and remyelination in the cuprizone animal model of MS. In the present study, we focus on the number of NG2+ cells and compare them with the number of APLNR-positive cells in the SVZ of three groups of animals—demyelination, remyelination, and control. In addition, we have incorporated the thickness of CC as a histomorphometric parameter for de- and remyelination and used confocal microscopy to assess the potential co-localization of APLNR expression in NG2+ cells. Lastly, in order to evaluate the memory and motor coordination of the animals, we carried out the passive avoidance and rotarod tests during the demyelination and remyelination periods and compared them with the results of the histomorphometric analysis of the CC and the results of the immunohistochemical and immunofluorescent assays.

## 2. Results

Histological examination—LFB/Cresyl violet.

**Figure 1:**
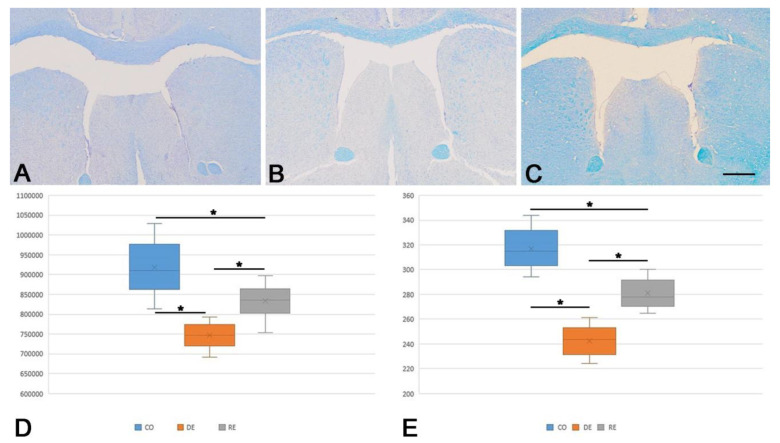
(**A**)—Luxol fast blue/Cresyl violet staining for visualization of the corpus callosum. Control group (CO); (**B**)—demyelination group (DE); (**C**)—remyelination group (RE). Scale bar—250 μm; (**D**)—graphical representation of the parameters area (μm^2^) and thickness (μm) of corpus callosum (**E**) in the control group (CO); demyelination group (DE); remyelination group (RE) presented with box and whisker plot showing the mean (x), surrounded by a ‘box’, the vertical edge of which is the interval between the lower and upper quartile [25–75%]. ‘Whiskers’ originating from this ‘box’ represent the non-outlier range. *—*p* < 0.001.

In order to histologically verify the demyelination and remyelination, we measured the thickness and area of the CC on LFB/Cresyl violet stained slides (Figure 1A–C). The highest thickness and largest area of the CC were measured in the control group. Both parameters decreased significantly after five weeks of cuprizone administration. On the other hand, five weeks after stopping the cuprizone, the area and thickness of the CC improved significantly, with the reported values being similar to those recorded in the control group. We conducted an ANOVA followed by post hoc analysis to assess differences among the three groups concerning the area and thickness of the CC. The ANOVA test revealed statistically significant differences between all groups. The subsequent post hoc analysis confirmed significant differences between each two pairs of examined groups (Figure 1D,E).

### 2.1. Immunohistochemistry

#### 2.1.1. APLNR Immunohistochemistry

**Figure 2:**
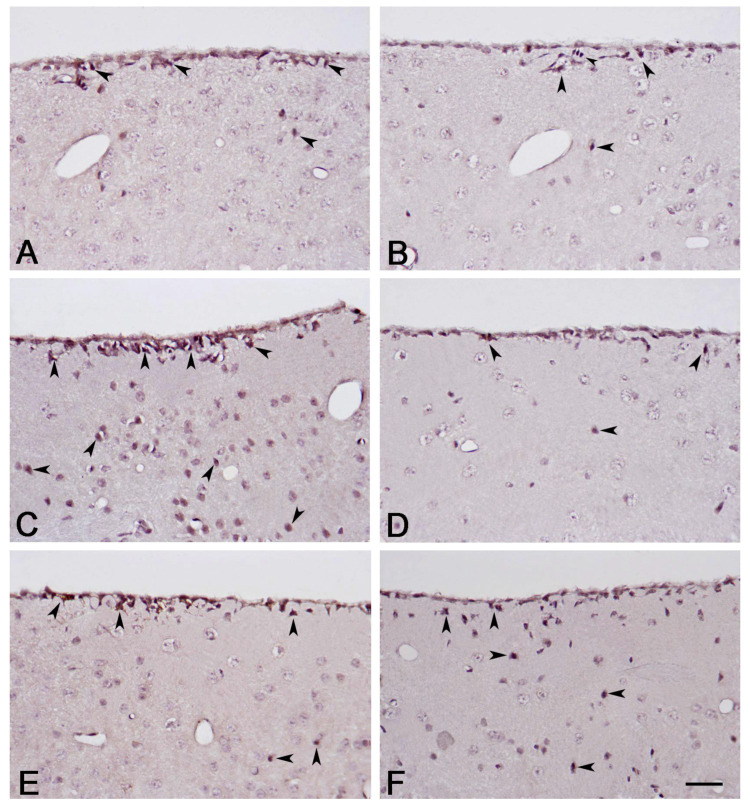
Immunohistochemical staining for apelin receptor (APLNR) in the control group (**A**); demyelination group (**C**); remyelination group (**E**). Immunohistochemical staining for neuronal/glial antigen 2 (NG2) in the control group (**B**); demyelination group (**D**); remyelination group (**F**). Scale bar—25 μm. Black arrow heads pointing positive cells.

The immunoreactivity of APLNR was visualized in the cytoplasm of various cells in the SVZ. Most positively stained cells were situated adjacently below the ependymal layer, and a lower number was observed in the deeper layers. Only in the demyelinating group were more cells observed in the deeper layers (Figure 2A,C,E). Significant changes in cell numbers were recorded. The lowest count was observed in the control group, where APLNR-positive cells averaged eight per field. A significant increase in APLNR-expressing cells was observed in the demyelination group, where we reported 18 cells per field on average. In addition, we reported a significant decrease in APLNR-stained cells in the remyelination group, where 11 cells per slide were observed on average.

#### 2.1.2. NG2 Immunohistochemistry

NG2+ cells were predominantly visualized under the ependymal layer, in a pattern of several adjacent cells arranged in a row or column. Individual cells were seldom visualized in deeper layers, a tendency predominantly noted in the de- and remyelination groups (Figure 2B,D,F). We quantified the number of NG2+ cells and reported significant changes in their count. On average, six NG2-expressing cells were visualized in the control group per field. The lowest count of NG2+ cells was recorded in the demyelination group, where the number of NG2+ cells averaged three cells per field, and, in some fields, no NG2+ cells were observed. In the remyelination group, we observed the highest count of NG2-expressing cells—12 per field on average.

### 2.2. Immunofluorescence Examination

**Figure 3:**
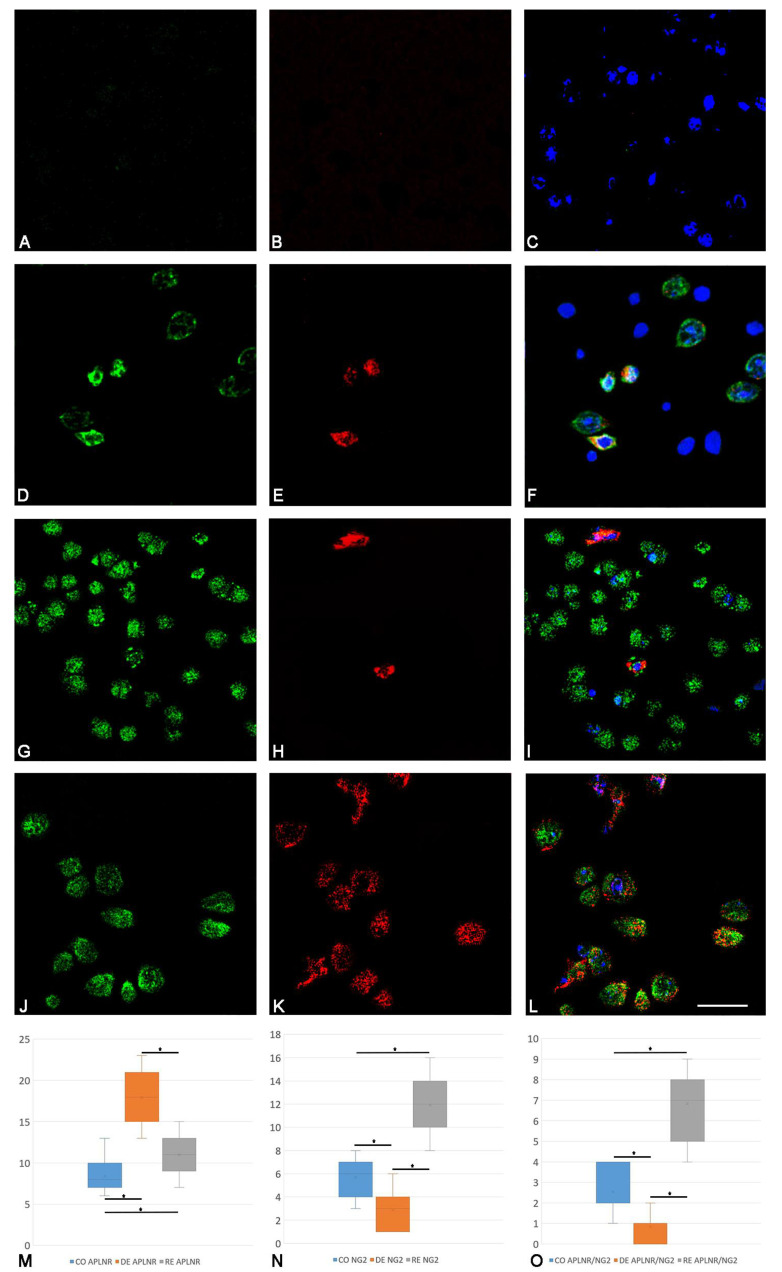
Immunofluorescence confocal microscopy. Green marks the apelin receptor (APLNR), red—neuro/glial antigen (NG2), blue—Hoechst for nuclear visualization; negative control—((**A**–**C**) Scale bar 20 μm); control group—((**D**–**F**) Scale bar 20 μm); demyelination group—((**G**–**I**) Scale bar 30 μm); remyelination group—((**J**–**L**) Scale bar 20 μm.). Graphical representation of the number of cells expressing APLNR (**M**); NG2 (**N**) and cells with co-localization of APLNR/NG2 (**O**), presented with box and whisker plot showing the mean (x), surrounded by a ‘box’, the vertical edge of which is the interval between the lower and upper quartile [25–75%]. ‘Whiskers’ originating from this ‘box’ represent the non-outlier range. CO—control group; DE—demyelination group; RE—remyelination group. *—*p* < 0.001.

Confocal microscopy confirmed our immunohistochemistry findings and enabled the quantification of cells co-expressing APLNR and NG2. In the control group, we observed an average of three co-localized cells per field, representing 46% of all NG2+ cells in this group (Figure 3A–C). In the demyelination group, co-localized cells were markedly reduced, with an average of one cell positive for both markers per field, accounting for 29% of all NG2+ cells (Figure 3D–F). Notably, some fields in this group lacked NG2+ cells entirely, resulting in the absence of cells showing co-localization in these fields. In contrast, the remyelination group exhibited the highest number of cells co-expressing APLNR and NG2, with an average of six cells per field, constituting 57% of all NG2+ cells in this group (Figure 3G–I).

We also tested for differences in the counts of APLNR-expressing cells, NG2-expressing cells, and cells co-expressing APLNR and NG2 among the control, demyelination, and remyelination groups. ANOVA results indicated significant differences across the three groups. Further analysis with a post hoc Tukey’s test revealed that all pairwise comparisons between groups were statistically significant, highlighting substantial group differences in mean cell counts. A summary of the data, including the number of cells expressing APLNR, NG2, and both markers, along with their statistical significance, is presented in Figure 3M–O.

**Figure 4:**
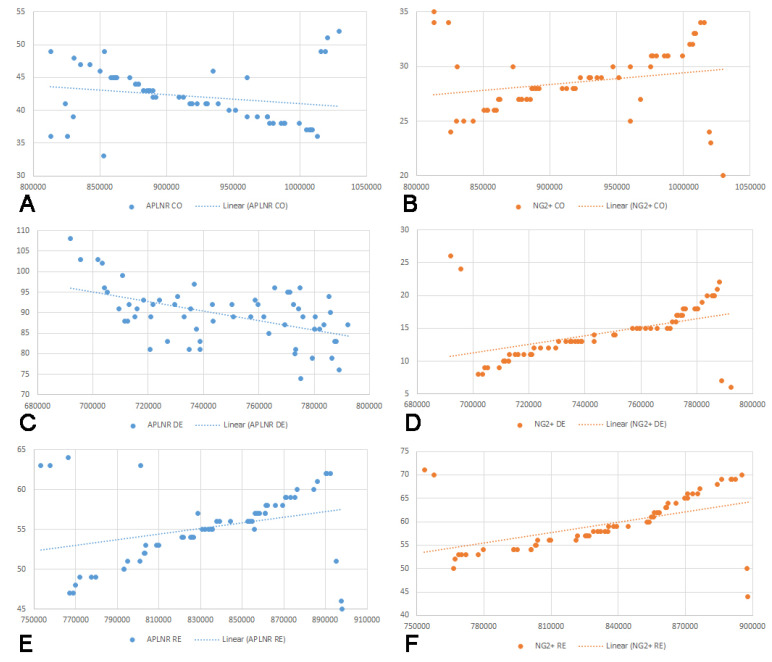
Graphical representation of correlations between the area of corpus callosum and the number of cells expressing apelin receptor (APLNR) in the control group (**A**), r = −0.21, *p* = 0.1; demyelination group (**C**), r = −0.52, *p* < 0.001; remyelination group (**E**) r = 0.31, *p* = 0.02. Graphical representation of correlations between the area of corpus callosum and the number of cells expressing neuronal/glial antigen 2 (NG2) in the control group (**B**) r = 0.22, *p* = 0.09; demyelination group (**D**) r = 0.46, *p* < 0.001; remyelination group (**F**) r = 0.39, *p* = 0.003.

**Figure 5:**
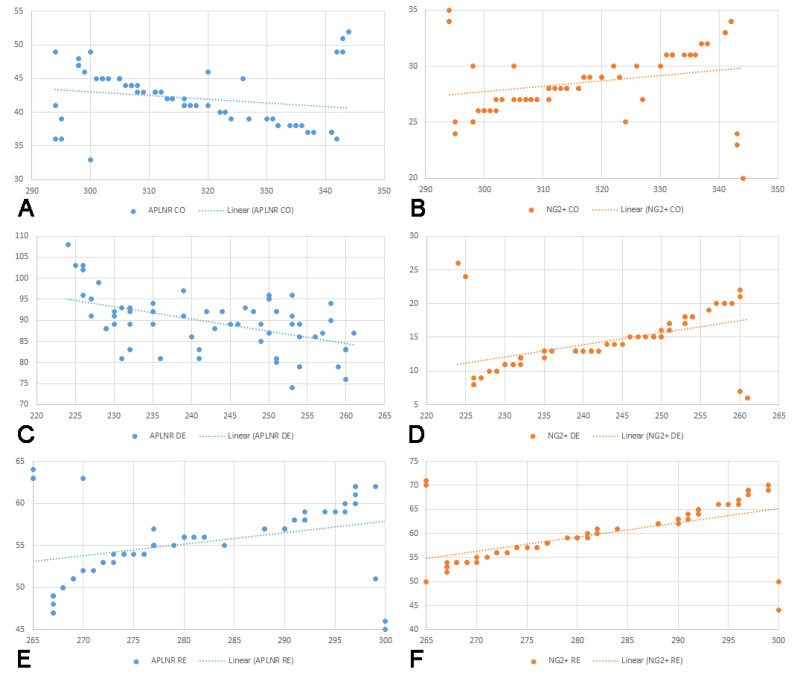
Graphical representation of correlations between the thickness of corpus callosum and the number of cells expressing apelin receptor (APLNR) in the control group (**A**) r = −0.20, *p* = 0.1; demyelination group (**C**) r = −0.50, *p* < 0.001; remyelination group (**E**) r = 0.34, *p* = 0.007. Graphical representation of correlations between the thickness of corpus callosum and the number of cells expressing neuronal/glial antigen 2 (NG2) in the control group (**B**) r = 0.25, *p* = 0.09; demyelination group (**D**) r = 0.48, *p* < 0.001; remyelination group (**F**) r = 0.41, *p* = 0.003.

Furthermore, we assessed whether correlations existed between the number of APLNR-positive cells and the area and thickness of the CC across the three experimental groups. A negative correlation was observed in the control group, though it was not statistically significant. Conversely, a statistically significant negative correlation was identified in the demyelination group. In the remyelination group, the correlation was positive, with moderate strength and statistical significance. Additionally, correlations were evaluated between the number of NG2+ cells and the CC area and thickness. All groups exhibited positive correlations; however, statistical significance was only observed in the demyelination and remyelination groups. The results of the correlation analysis are graphically represented in Figure 4 and Figure 5.

### 2.3. Behavior Tests

**Figure 6:**
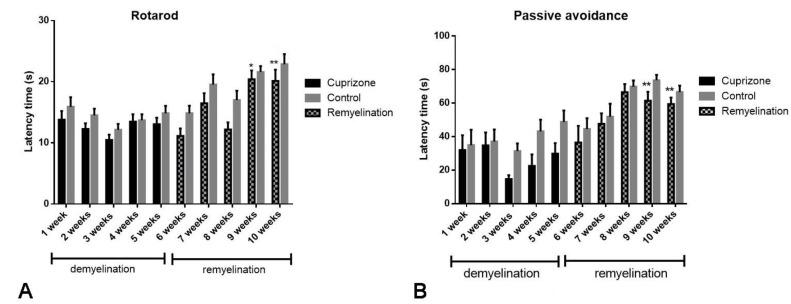
Graphical representation of the results of the performed behavior test. (**A**)—The rotarod test performed once weekly during the demyelination and remyelination period. The results are presented as mean ± SEM. * *p* < 0.05—Statistically significant improvement in motor coordination between weeks 2 and 9, ** *p* < 0.01—between weeks 3 and 10 in the demyelination and remyelination group; (**B**)—the passive avoidance test performed once weekly during the demyelination and remyelination period. The results are presented as mean ± SEM. ** *p* < 0.01—statistically significant increase in latency time was observed between weeks 3 and 9, 3 and 10 when comparing periods of demyelination and remyelination.

To evaluate the effects of cuprizone intake on motor coordination and memory in the experimental animals, we conducted two behavioral tests: the rotarod test to assess motor coordination and the passive avoidance test to evaluate memory. The tests were performed once a week. The mean latency time in the rotarod test was 17 s for the control group, 12 s for the demyelination group, and 15 s for the remyelination group. We used the Mann–Whitney U test to analyze statistically significant differences in latency times. The results showed a significant decrease in latency times during weeks 1–5 of the demyelination phase compared to the control group (*p* < 0.001) and a significant improvement in the remyelination group (weeks 6–10) compared to the demyelination phase (weeks 1–5) (*p* < 0.001). Additionally, a comparison between the remyelination phase (weeks 6–10) and the corresponding period in the control group also showed a statistically significant difference (*p* = 0.003). To capture more detailed trends, we further analyzed changes in latency time on a week-by-week basis across all phases. In the demyelination period, the motor coordination of the experimental animals worsened between the first and third weeks. Afterward, during the fourth and fifth weeks, we observed a slight improvement in balance, and during the remyelination period, a gradual improvement in the balance of the mice was registered, especially in weeks ninth and tenth (Figure 6A). We reported a statistically significant improvement in motor coordination between the second and ninth week (*p* < 0.05) and between the third and tenth week (*p* < 0.01).

The results of the passive avoidance test show a similar tendency, as the mean latency time for the control group was 49 s, 28 s for the demyelination group, and 44 s for the remyelination group. The Mann–Whitney U test revealed a significant decrease in latency times during weeks 1–5 of the demyelination phase compared to the control group (*p* < 0.001) and a significant improvement in the remyelination group (weeks 6–10) compared to the demyelination phase (weeks 1–5) (*p* < 0.001). Additionally, a comparison between the remyelination phase (weeks 6–10) and the corresponding period in the control group showed a statistically nonsignificant difference (*p* = 0.14). The week-by-week analysis showed similar results to the Rotarod test. Between the 1st and 3rd week of the experiment, we observed a decrease in the latency time in the Cuprizone group, which indicates learning and memory decline. At the end of the demyelination period and at the beginning of the remyelination period, we registered a gradual increase in the latency time. The most prominent improvement in memory was observed throughout the last three weeks of the experiment (Figure 6B). A statistically significant increase in latency time was observed between weeks 3 and 9 (*p* < 0.01) and between the 3rd and 10th week (*p* < 0.01) when comparing periods of demyelination and remyelination.

**Figure 7:**
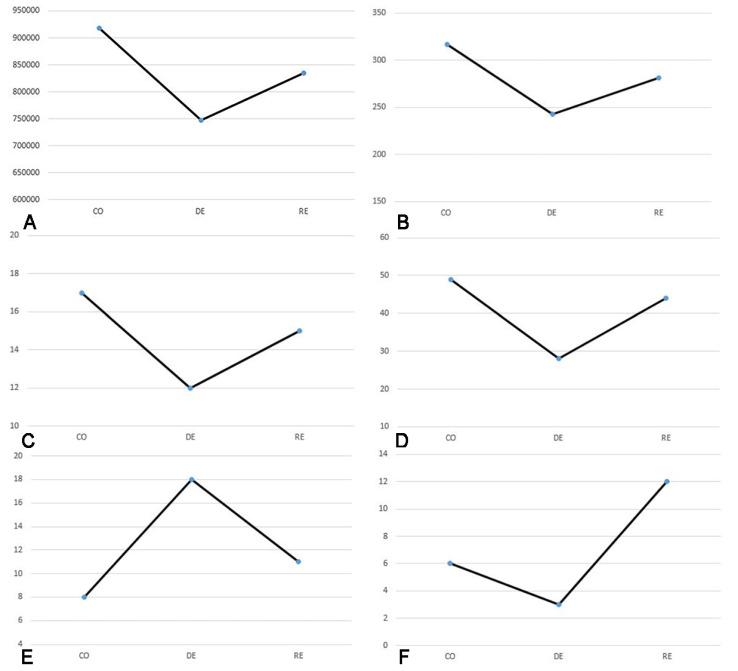
Graphical representation of changes in mean values for (**A**) corpus callosum area (μm^2^), (**B**) corpus callosum length (μm), (**C**) latency time in the rotarod test (s), (**D**) latency time in the passive avoidance test (s), and number of (**E**) APLNR-positive cells and (**F**) NG2-positive cells in the subventricular zone across control (CO), demyelination (DE), and remyelination (RE) groups.

To evaluate the alignment between behavioral test outcomes and the histomorphometric analysis of the CC, we compared trends in the mean CC area and thickness with mean values from the rotarod and passive avoidance tests across the control, demyelination, and remyelination groups. Additionally, to assess the relationship between immunohistochemical and immunofluorescence findings and behavioral performance, we analyzed trends in the mean counts of APLNR- and NG2-positive cells in the SVZ alongside changes in the mean values from the rotarod and passive avoidance tests across these three groups (Figure 7).

## 3. Discussion

In the present study, we performed an extensive analysis of the number of the APLNR- and NG2-expressing cells in the anterior SVZ of a mouse model during the processes of demyelination and remyelination in the cuprizone model. The pertinent literature suggests that the CC is a primary target of acute demyelination after cuprizone administration, followed by partial remyelination if the cuprizone is removed from the diet [5,47]. To assess the changes in the CC, we measured its thickness and area in mice after five weeks of cuprizone administration and after five more weeks without it on LFB/cresyl violet stained slides. Our results demonstrated significant differences in both the area and thickness of CC, as the recorded values were the lowest in the demyelination group, higher in the remyelination group, and the highest in the control group. The literature data suggests that demyelination is based on the rapid loss of oligodendrocytes, which is on par with our data where the morphometric values of CC after cuprizone treatment are significantly lower compared to the control group [5,8]. In recent studies, the remyelination in the CC is connected with the migration and differentiation of new mature oligodendrocytes [48]. Our study demonstrates the depletion of NG2+ cells in the SVZ after 5 weeks of cuprizone-induced demyelination, which aligns with the suggested migration of progenitor cells by Leo et al. [48]. Moreover, the recorded increase in the thickness and area of CC in the remyelination group further supports the theory of NG2+ migration and proliferation in CC [48]. The aforementioned observations show that LFB/cresyl violet staining, followed by morphometric analysis of CC in mice models of demyelination and remyelination, is a reliable way to evaluate the ongoing changes.

APLNR is a G-protein coupled receptor, widely distributed in different mammalian groups, including rats, mice, and humans [49,50]. Apelin and elabela are two different endogenous ligands for APLNR described in multiple mammals, including humans [51,52,53,54]. While elabela is commonly connected to embryonic heart development, multiple studies describe apelin distribution in the human CNS [52,53,54,55]. Even though apelin and APLNR mRNA are detected in the CNS, the actual distribution of the receptor is poorly described, and it is yet to be fully understood [52]. APLNR and apelin expression are functionally linked to neurogenesis, suppressing neuroinflammation, neovascularization, and proliferation [38,56,57,58]. Because of this, we conducted an immunohistochemical staining for APLNR in the SVZ, which is referred to as a germinal region in the brain, aiming to study changes in APLNR expression levels and its potential role in the process of demyelination and remyelination [15].

To the best of our knowledge, we are the first to demonstrate a significant increase in the expression levels of APLNR in the SVZ of mice after five weeks of cuprizone administration compared to the control group. A potential explanation for these data is the fact that demyelination is always linked to inflammation [59]. Therefore, the rise in APLNR could be an adaptive mechanism aimed at modulating the inflammatory reaction. This hypothesis is also supported by the correlation analysis performed between APLNR expression and the morphometric values of the CC. Both the area and thickness of the CC were reported to correlate negatively with APLNR expression, and those correlations were statistically significant. Therefore, more severe demyelination is coupled with an increase in APLNR expression, which might suggest a potential involvement of APLNR in countering the ongoing demyelination in the cuprizone-treated mice. This statement is further strengthened by our data, where the expression of APLNR decreased significantly in the remyelination group compared to the demyelination group, yet remained higher than in the control groups. It can thus be assumed that after the acute demyelination and inflammation phase, APLNR levels start to return to normal following the remyelination process. Even though the overall expression of APLNR was significantly lower in the remyelination group, the correlation analysis with the area and thickness of CC revealed a moderately positive correlation, showing better morphometric values of CC, and suggests that higher APLNR expression is followed by better remyelination.

NG2+ cells are progenitor cells found in multiple brain areas, including the SVZ, which can differentiate into oligodendrocytes and other glial cell types [20,21]. Based on this, we performed immunohistochemical staining to visualize NG2+ cells in the SVZ and evaluate their changes following demyelination and remyelination in our experimental model. Our results showed a decrease in the number of NG2+ cells in the SVZ after five weeks of cuprizone administration compared to the control group. Previous studies have suggested an increase in the number of NG2+ in the CC as an adaptive mechanism to compensate for the loss of oligodendrocytes [60]. The observed decrease in NG2+ cells during the demyelination period of our study could be explained by several factors: direct toxicity of cuprizone on NG2+ cells, migration of these cells to demyelination sites followed by differentiation into oligodendrocytes, or a combination of both. The performed correlation analysis between the number of NG2+ cells and the morphometric values of CC revealed a positive correlation, implying that the higher NG2+ cell count corresponds to higher values of the area and thickness of the CC. A plausible explanation might be that the SVZ, as a germinal zone, reacts to changes in the CC [14]. Another possible explanation would be the direct toxicity of cuprizone. It is likely that the low values for the area and thickness of the CC are related to the more severe effects of cuprizone, which can be associated with higher toxicity and more extensive cell death. Conversely, higher morphometric values of the CC can be linked to lower toxicity of cuprizone and, overall, less pronounced cell death, including in the SVZ. Our findings are further supported by the drastic increase in the number of NG2+ cells in the remyelination group, where their count is doubled compared to the control group and quadrupled compared to the demyelination group. Our results showed that the removal of the toxic agent is followed by the intensive proliferation of NG2+ cells in the SVZ of the remyelination group. The observed increase in the NG2+ cell count aligns with the enlargement in both the area and thickness of CC. This finding is consistent with previous studies that describe NG2+ cells as a primary reservoir for new oligodendrocytes. [61]. From the correlation analysis in the remyelination group, we observed that the higher morphometric values of the CC correlated with a higher NG2+ cell count in the SVZ, which goes in the same line of logic, where an increased NG2+ cell population is directly related to a more prominent remyelination and better recovery in mice.

As part of the present study, we performed confocal microscopy to demonstrate the presence of NG2+ cells expressing APLNR. This co-localization is previously mentioned in the literature but not in the context of demyelination and remyelination [62]. Our results in the control group demonstrated that around half of the NG2+ cells in the SVZ expressed APLNR, while in the mice treated with cuprizone for 5 weeks, this co-localization decreased to less than one-third. On the other hand, the overall expression of APLNR was significantly higher in mice exposed to five weeks of cuprizone treatment. This leads to the assumption that APNLR peak expression levels during cuprizone administration and demyelination are not related to increased expression by NG2+ cells. Other cell types expressing APLNR are well documented in the literature, such as microglial cells, mesenchymal-like cells, and astrocytes [62,63]. They might potentially be responsible for the rise in APLNR expression in cuprizone-treated animals. While this could be a potential cornerstone in future experiments, we suggest that the demonstrated downregulation in the expression of APLNR by NG2+ cells in the mice post 5 weeks of cuprizone treatment is due to the overall decrease in their number in the SVZ. On the contrary, in the remyelination group, we observed a drastic increase in the co-localization of NG2+ cells and APLNR to almost two-thirds. This strengthens the hypothesis of the close relationship between APLNR and NG2+ cells during remyelination. Bearing in mind that the overall APLNR expression goes down compared to the demyelination group, we assume that NG2+ cells dominate over all other cell types expressing APLNR and are the primary source of the relatively high levels of APLNR expression in the remyelination group. Given the previously observed positive correlations between the morphometric values of CC with APLNR expression on one hand and with NG2+ cells on the other, we propose that APLNR expression might play a potential role in the observed remyelination in our experimental model.

In addition to morphological assessments, we conducted two behavioral tests—the rotarod test, which evaluated motor coordination and balance, and the passive avoidance test, which assessed memory function. These tests aimed to identify potential behavioral alterations associated with cuprizone-induced demyelination and the subsequent remyelination phases. By comparing behavioral outcomes with morphological changes observed in the brain, we sought to determine whether the demyelination and remyelination processes were paralleled by functional impairments or recoveries in motor and cognitive abilities. Our results revealed that during the demyelination phase, both motor coordination and memory declined significantly, reaching their lowest levels in the fifth week of cuprizone administration. Specifically, the mean latency time in the rotarod test decreased from 17 s in the control group to 12 s in the demyelination group, with a partial recovery to 15 s in the remyelination group. Similarly, the passive avoidance test revealed a decline in latency time from 49 s in the control group to 28 s in the demyelination group, with an improvement to 44 s in the remyelination group. The results of the behavioral tests showed a trend that mirrors the findings from the histomorphometric analysis of the CC. These behavioral changes were paralleled by alterations in the CC morphology. The mean area of the CC was 917,419 µm^2^ in the control group, decreased to 747,343 µm^2^ during demyelination, and partially recovered to 834,356 µm^2^ in the remyelination group. Similarly, the thickness of the CC decreased from 317 µm in the control group to 243 µm during demyelination, with recovery to 281 µm in the remyelination phase. Together, these findings indicate a significant decrease in the CC integrity during demyelination, followed by a substantial but incomplete restoration during remyelination, consistent with the observed trends in motor coordination and memory performance.

Many reports have suggested that the apelin/APLNR system may be a potential target for regulating learning and memory processes, and a variable impact on memory may be obtained due to different experimental conditions or animals used [64]. The study by Han et al. (2014) revealed that an intracerebroventricular injection of apelin-13 impairs short- and long-term memory in mice in a novel object recognition test [64]. On the other hand, another study showed that apelin-13 promoted memory consolidation in mice during passive avoidance tasks [65]. Our study reviewed memory deterioration during cuprizone administration within the third, fourth, and fifth weeks of the experiment. At the end of the demyelination period and the beginning of the remyelination period, we registered a slight increase in the latency time, suggestive of memory improvement. Overall our results suggest a significant deterioration in memory in the demyelination group compared to controls and a substantial improvement in the remyelination group. Meanwhile, we recorded a significant increase in APLNR-expressing cells in the demyelination group, where we reported 18 cells per field on average. The most prominent improvement in memory was observed throughout the last three weeks of the experiment in the passive avoidance test when a significant decrease in the number of APLNR-stained cells in the remyelination group was observed. The lowest count of APLNR-positive cells was observed in the control group, where a gradual increase in the latency time was registered from the first to the tenth week. Apart from memory, we also assessed the motor coordination of the experimental animals in the three groups. To our knowledge, we are the first to report an association between motor function and APLNR expression in the SVZ of cuprizone-treated mice. Similarly, for the effects on memory, we report a significant decrease in the latency time of the rotarod test in the demyelination group, which improved in the remyelination group, nearly reaching control levels. In essence, our results display an opposite trend between the results of the behavior test and the count of APLNR-positive cells across the experimental groups. While behavioral performance was the highest in the control group, declined during demyelination, and partially improved in the remyelination phase, APLNR expression was the lowest in the control group, peaked in the demyelination group, and decreased in the remyelination phase but remained above the control levels. Our experiment demonstrated that both memory and motor improvements were associated with fewer APLNR-positive cells.

The NG2+ cell count showed a different pattern: the lowest levels were observed during demyelination (mean count of 3), the highest during remyelination (mean count of 12), and intermediate levels in the control group (mean count of 6). This follows the same trend as the comparison of behavior tests and morphometric values of the CC. Even though these apparent similarities suggest a connection between the NG2+ cells and the data gathered from the behavior tests, no major conclusions can be drawn, especially considering the fact that we performed an analysis of NG2+ cells only in the SVZ. Further experiments in this field may prove useful for a better understanding of NG2+ cells’ role in motor and memory changes during demyelination and remyelination.

The present study has several limitations that warrant consideration. First, the cuprizone mouse model used in this research, particularly the oligodendrocyte loss it induces, requires further investigation to fully understand its mechanisms and relevance to human pathology. Additionally, all mice used in this study were male, a choice made to eliminate potential confounding effects of female sex hormones and their periodic fluctuations. However, this limits the generalizability of the findings across both sexes, as the potential influence of sex differences on the observed outcomes was not explored. It is also important to note that our evaluation of APLNR and NG2+ cell counts was restricted to the SVZ, given its role as a primary germinal region. For a more comprehensive understanding, however, APLNR expression should be assessed across additional brain regions. Expanding this analysis is essential before drawing substantial conclusions about the broader role of APLNR during de- and remyelination in the cuprizone model. Moreover, this study focuses exclusively on the morphological and behavioral changes observed in the cuprizone model of MS, and additional steps are necessary to draw meaningful conclusions applicable to human MS. The findings presented here must be validated in other experimental MS models to establish their robustness and reliability before any potential human application can be considered. Further studies are essential to address these gaps and enhance the broader applicability of our results. Nonetheless, this study provides a foundational basis for future research, since it offers valuable data regarding the de- and remyelination occurring in the cuprizone animal model.

## 4. Materials and Methods

### 4.1. Experimental Animals

Thirty 8-week-old male C57BL/6 mice were provided by the Vivarium of the Faculty of Medicine at the Medical University of Sofia, Bulgaria. Male mice were randomly divided into three groups: a control group, a demyelination group, and a remyelination group. The control group consisted of 10 male mice maintained on a standard diet without cuprizone administration. The demyelination group consisted of 10 male mice subjected to a 5-week diet supplemented with 0.2% cuprizone. The remyelination group, 10 male mice, also underwent a 5-week cuprizone diet for the initial induction of demyelination, followed by a 5-week recovery phase in which cuprizone was removed from the diet. For the duration of the experimental study, the animals were kept under controlled conditions: room temperature, 22 ± 3 °C; humidity, 30%; lighting schedule, 12 h light/dark cycle. Experiments were performed during the light part of the cycle. All animal experiments were carried out in accordance with Directive 2010/63/EU of the European Parliament and of the Council on protecting animals used for scientific purposes and was approved by the Bulgarian Food Safety Agency (Approval Protocol No. 361 of 24 October 2023).

### 4.2. Cuprizone-Induced Demyelination

The demyelination was performed by administering the neurotoxic agent cuprizone to 8-week-old C57BL/6 mice for five weeks. The method is well characterized and has different modifications [7,44]. Cuprizone (CAS: 370-81-0, Sigma Aldrich, Vienna, Austria) was administered with drinking water at a concentration of 0.2% (*w*/*v*). After five weeks, cuprizone intake was discontinued, and the mice received only drinking water for the next five weeks to allow remyelination. The control group received only drinking water throughout the experiment.

### 4.3. Tissue Preparation

The experimental animals were anesthetized using an intraperitoneal injection of thiopental sodium (Sigma Aldrich Chemie GmbH, Taufkirchen, Germany) (30 mg/kg body weight) and then perfused transcardially with 4% paraformaldehyde (CAS: 30525-89-4, Sigma-Aldrich, Vienna, Austria) in 0.1 M phosphate-buffered saline (Merck Catalogue No. 1465920006) at pH 7.4. The brains (30 in total, with 10 from each group) were subsequently isolated and fixed in the same solution for 24 h. The tissues were rinsed, dehydrated, and embedded in paraffin blocks (Merck Catalogue No. 1071511000) after fixation. Serial coronal sections, each 6 μm thick (from bregma 0.38 to bregma 0.62 according to the Mouse Brain Atlas by Paxinos and Franklin [66]), were prepared using a paraffin microtome (Leica RM 2155, Wetzlar, Germany).

### 4.4. Histological Techniques

Tissue sections were mounted on gelatin-coated slides, deparaffinized, rehydrated to 95% alcohol (Merck Catalogue No. 1009835000), and incubated in a 0.01% Luxol fast blue (LFB) solution (CAS: L0294, Sigma-Aldrich, Vienna, Austria) for 6 h at 58 °C. The sections were then differentiated in a 0.05% lithium carbonate solution (CAS: 255823, Sigma-Aldrich, Vienna, Austria) and counterstained with Cresyl violet (CAS: 41830-80-2, Sigma-Aldrich, Vienna, Austria), resulting in myelinated fibers appearing blue, the neuropil pink, and the nerve cells purple. After drying for 24 h, the sections were coverslipped with Entellan (Merck Catalogue No. 1079600500). Every fifth coronal section of each brain (bregma 0.38–0.62) was examined and documented using an Olympus CX 21 microscope fitted with an Olympus C5050Z digital camera (Olympus Optical Co., Ltd., Tokyo, Japan) at 40× magnification.

### 4.5. Immunohistochemistry

After deparaffinization in three changes in xylene (Merck Catalogue No. 1082984000) for 10 min each, the sections were rehydrated in a descending alcohol series, 100%, 95%, 90%, 80%, 70% ethanol (Merck Catalogue No. 1009835000), and distilled water for 5 min each. This was followed by antigen retrieval by heating the slides at 95 °C in Citrate Plus (10×) HIER Solution (pH 6.0) (Cat. No. CPL500, ScyTek Laboratories Inc., Logan, UT, USA) for 20 min. After cooling and three washes in Tris-Buffered Saline with 0.05% Tween 20 (TTBS) (E-BC-R335, Wuhan Elabscience Biotechnology Co., Ltd., Hubei Sheng, China) for 5 min each, endogenous peroxidase was blocked with 3% H_2_O_2_ in distilled water for 10 min, followed by three washes in TTBS for 5 min each. The sections were then incubated with Super Block (ScyTek Laboratories Inc., Logan, UT, USA) for 5 min at room temperature. To block endogenous biotin, the appropriate kit (Cat. No. BBK120, ScyTek Laboratories Inc., Logan, UT, USA) was used. The sections were incubated according to the kit instructions: 15 min with part A, washing, and 15 min with part B. Blocking of endogenous mouse immunoglobulins was performed with a mouse-to-mouse blocking reagent (Cat. No. MTM015, ScyTek Laboratories Inc., Logan, UT, USA) for 1 h at RT. After washing in TTBS, the sections were incubated with the following primary antibodies: polyclonal anti-APLNR antibody (E-AB-13919—Wuhan Elabscience Biotechnology Co., Ltd., Hubei Sheng, China) and mouse monoclonal anti-NG2 antibody (SC-53389, Santa Cruz Biotechnology Inc., Dallas, TX, USA), marking oligodendrocyte progenitors, overnight at 4 °C (dilution 1:200). After incubation, the immunoreactivity of APLNR and NG-2 was visualized with UltraTek HRP Anti-Polyvalent Staining System (Cat. No. AFN600, ScyTek Laboratories Inc., Logan, UT, USA) according to the manufacturer’s instructions. Sections were counterstained with hematoxylin, dehydrated in graded series of ethanol, cleared in xylene, and cover-slipped. For controls, the primary antibody was replaced with antibody diluent.

### 4.6. Immunofluorescence

After deparaffinization in three changes in xylene (Merck Catalogue No. 1082984000) for 10 min each, the sections were rehydrated in a descending alcohol series, 100%, 95%, 90%, 80%, 70% ethanol (Merck Catalogue No. 1009835000), and distilled water for 5 min each. This was followed by antigen retrieval by heating slides at 95 °C in Citrate Plus (10×) HIER Solution (pH 6.0) (Cat. No. CPL500, ScyTek Laboratories Inc., Logan, UT, USA) for 20 min. After cooling and three washes in Tris-Buffered Saline with 0.05% Tween 20 (TTBS) (E-BC-R335, Wuhan Elabscience Biotechnology Co., Ltd., Hubei Sheng, China) for 5 min each, the sections were incubated with Super Block (ScyTek Laboratories Inc., Logan, UT, USA) for 5 min at room temperature. The blocking of endogenous mouse immunoglobulins was performed with a mouse-to-mouse blocking reagent (Cat. No. MTM015, ScyTek Laborato-ries Inc., Logan, UT, USA) for 1 h at RT. After washing three times in TTBS for 5 min each, the sections were incubated with the following primary antibodies: polyclonal anti-APLNR antibody (E-AB-13919, Wuhan Elabscience Biotechnology Co., Ltd., Hubei Sheng, China) and mouse monoclonal anti-NG2 antibody (sc-53389—Santa Cruz Biotechnology Inc., Dallas, TX, USA) overnight at 4 °C (dilution 1:200). After incubation, slides were washed in TTBS and incubated with secondary antibodies: Goat Anti-Rabbit IgG (H + L) (Elab Fluor^®^ 488 conjugated) (E-AB-1055, Wuhan Elabscience Biotechnology Co., Ltd., Hubei Sheng, China) and Goat Anti-Mouse IgG (H + L) (Elab Fluor^®^ 594 conjugated) (E-AB-1059, Wuhan Elabscience Biotechnology Co., Ltd., Hubei Sheng, China) for 1 h at room temperature. After three washes in TTBS for 5 min each, the sections were incubated with Hoechst 33342 (sc-391054, Santa Cruz Biotechnology Inc., Dallas, TX, USA) for 10 min, then washed three times in TBS (E-IR-R116, Wuhan Elabscience Biotechnology Co., Ltd., Hubei Sheng, China) for 5 min each. The sections were then mounted with FluoreGuard Mounting Medium (Hard Set) (FMH030—ScyTek Laboratories Inc., Logan, UT, USA), coverslipped, and kept away from light at 4 °C. The slides were observed on a Leica TCS SPE confocal microscope (Wetzlar, Germany).

### 4.7. Statistical Analysis

Statistical analysis and graphical presentation of the results were performed with the program GraphPad Prism 6.0 and SPSS software version 28.0.0.1 (IBM Corporation, Armonk, NY, USA). The arithmetic means and the standard errors of the arithmetic mean (SEM) were determined for all data. The presence of statistically significant differences between the compared means was assessed using the Mann–Whitney U test, one-way ANOVA, and Tukey’s test. A *p*-value of 0.05 or lower was considered statistically significant. Ten animals were sacrificed from each group (control, demyelination, and remyelination), and their brains were collected for analysis. Six slides were prepared per group, and five fields per slide (n = 300) were examined to count the number of immunopositive cells for APLNR, NG2, and their co-localization. The methodology used for the cell counts followed the established protocol by Jin et al. [67]. Normality of data distribution was evaluated using the Kolmogorov–Smirnov test, which indicated a non-normal distribution. To identify significant differences in the number of cells expressing APLNR, NG2, and their co-localization, we used analysis of variance (ANOVA) followed by Tukey’s Honest Significant Difference (HSD) post hoc test. Spearman’s correlation analysis was conducted to examine the relationships between the area and thickness of the CC per slide and the number of cells expressing APLNR, NG2, and their co-localization, quantified as the total number of cells across five examined fields per slide. A standard significance threshold of α (*p*-value) = 0.05 was used for all statistical tests.

### 4.8. Behavior Tests

#### 4.8.1. Passive Avoidance Test

The test was performed in an automated active and passive avoidance system (Gemini Avoidance System, San Diego, CA, USA). The apparatus comprises two identical compartments (25cm × 20cm × 16 cm) with an electrified grid floor. The chambers are divided by a wall with a guillotine door (8 cm × 6 cm). Each mouse was placed in the lighted chamber, and when it entered the dark chamber, a weak electric shock with an intensity of 0.5 mA (3 s) was applied through the grid floor. The degree of learning and memory of experimental animals was estimated by the latency period—the time needed for entering the dark chamber. The maximal latency period for every trial was 300 s. The test was conducted once weekly throughout the whole experiment [44].

#### 4.8.2. Rotarod Test

The rotarod test is used to evaluate motor coordination in mice and rats. The rotarod apparatus (Ugo Basile, RotaRod 47 600) comprises a rotating cylinder divided into different compartments with opaque walls, one for each animal. Mice were placed on the rod of the apparatus in an accelerating mode from 4 to 40 rpm (acceleration time 30 s), and the latency to fall was recorded. Each animal had three consecutive trials with an inter-trial interval of 15 min. The average time of the three consecutive trials was used for the analysis [68]. The maximum duration of every trial was 300 s. The rotarod test was performed once weekly throughout the whole experiment.

## 5. Conclusions

In conclusion, our study highlights pronounced histomorphological and behavioral changes during both demyelination and remyelination in the cuprizone animal model. Specifically, we observed a significant reduction in the area and thickness of the CC in the demyelination group, followed by a marked recovery in these measurements during remyelination, although the values did not fully return to control levels. Behavioral testing similarly revealed substantial impairments in motor coordination and memory during demyelination, with partial improvement observed during remyelination, though still below the performance of healthy controls. We also demonstrate significant alterations in the number of APLNR and NG2+ positive cells in the SVZ. APLNR-positive cells peaked in the demyelination phase and gradually decreased during remyelination, yet remained elevated compared to controls. In contrast, the NG2+ cell counts were the lowest in the demyelination group and the highest in the remyelination group. Additionally, our data demonstrated a significant co-localization of NG2+ and APLNR-expressing cells within the SVZ, with the highest count of NG2+/APLNR cells occurring during remyelination and the lowest during demyelination, mirroring the overall NG2+ cell trends. These findings provide a morphological basis for understanding the role of APLNR and NG2+ cells in the SVZ during demyelination and remyelination in the experimental cuprizone animal model. However, further studies are essential to investigate APLNR expression in other brain regions to fully elucidate its role. Additionally, further validation in alternative experimental models is required before translating these findings to the pathogenesis and potential treatment strategies for MS.

## Data Availability

The raw data supporting the conclusions of this article will be made available by the authors upon request.
